# Chemical composition, antioxidant activity, and diuretic effect of Moroccan fresh bee pollen in rats

**DOI:** 10.14202/vetworld.2020.1251-1261

**Published:** 2020-07-03

**Authors:** Asmae El Ghouizi, Nawal El Menyiy, Soraia I. Falcão, Miguel Vilas-Boas, Badiaa Lyoussi

**Affiliations:** 1 Laboratory of Natural Substances, Pharmacology, Environment, Modeling, Health and Quality of Life (SNAMOPEQ), University of Sidi Mohamed Ben Abdellah, Fez 30 000, Morocco; 2 Centro de Investigação de Montanha, Instituto Politécnico de Bragança, Campus de Santa Apolónia, 5300-253 Bragança, Portugal

**Keywords:** antioxidant activity, diuresis, fresh bee pollen, liquid chromatography–mass spectrometry, phenolamides

## Abstract

**Aim:**

This study investigated the chemical composition, antioxidant activity, and diuretic effect of Moroccan aqueous extract of fresh bee pollen (AEFBP) in normal rats.

**Materials and Methods:**

The chemical composition of the extracted bioactive compounds was assessed using liquid chromatography with diode array detection coupled to electrospray ionization (ESI) tandem mass spectrometry (LC/DAD/ESI-MS^n^). 2,2-diphenyl-1-picrylhydrazyl and the reducing power were used to assess the antioxidant properties of the extract, together with the determination of total phenols and flavonoids. To assess the diuretic effect, 20 normal rats were divided into five groups: The first was a control group administered by distilled water (10 mL/kg body weight), the second group received furosemide (10 mg/kg body weight), the third group received 100 mg/kg body weight of AEFBP, the fourth group received 250 mg/kg body weight of AEFBP, and the fifth group received 500 mg/kg body weight of AEFBP for 30 days. Toward the end of this experiment, urine output was measured, and plasma and urine were sampled to analyze creatinine, potassium, chloride, and sodium levels.

**Results:**

*N*^1^,*N*^5^,*N*^10^-tri-p-coumaroylspermidine is a spermidine derivative and was the main compound in this sample, in a total of 19 compounds identified, including flavonoids, glucoside flavonoids, and methylated derivatives. Force feeding with the AEFBP induced a significant increase in urine output and urinary electrolyte levels with a dependent dose-effect without changes in plasma electrolytes, whereas furosemide decreased plasma potassium.

**Conclusion:**

Moroccan fresh bee pollen extract contains flavonols and spermidines that induce a potential antioxidant activity related to significant diuretic effect without changes in plasma composition.

## Introduction

History of apitherapy and hive products dates back to the old times, being used in phytotherapy and in diet due to their powerful healing properties [1]. At present, apicultural products (bee honey, royal jelly, propolis, bee wax, bee bread, and bee pollen) attract a particular interest from the scientific committee because of their rich and varied composition of bioactive molecules that have a strong beneficial properties to human health [2,3]. Bee pollen is defined as the flower’s male gametophyte collected by worker honeybees (*Apis* sp. including *Apis mellifera*) and stingless bees and mixed with nectar’s sugar, enzymes, wax, and their pharyngeal substances to bind the grains and compact the powder [4]. Thereafter, the collected ­pollen powder is carried as a small pellet by the bee’s legs to their hive, where it will be gathered, kept, and consumed as bee food for all the growth process in the hive [5,6]. In general, the fresh pollen contains 21-30% of water, which requires quick freezing to preserve its nutritional value, often assessed through concentration of protein, sugars, carbohydrates, vitamins, lipids, and phenolic compounds [1,7]. These compounds are considered among the most bioactive molecules because of their antioxidant power. Pollen grains, especially the pollen coat, present an accumulation of tri-substituted spermidines, which are N-acylated biogenic amines conjugated with phenolic acids, which are essentially hydroxycinnamoyl acids [8]. These secondary metabolites are involved in defense responses, development, and reproduction associated with pollinator attraction [9]. Spermidine amides are well distributed in pollens of Rosaceae, Betulaceae, Fagaceae, and Juglandaceae families and constitute a useful taxonomic marker in higher Hamamelidae [10].

High blood pressure is a major global public health problem considering the increasing number of patients affected every year [11]. Moreover, low efficacy and adverse effects associated with synthetic diuretics (antihypertensive drugs) may limit the use of these drugs, resulting in recommending a concurrent use of an adequate regimen [12]. In this context, several studies have been conducted to assess the beneficial effect of food diet and natural products on hypertension and associated pathologies. All of these studies support that adopting a diet naturally rich in essential elements, vitamins, and antioxidants can have a strong effect on preventing and managing high blood pressure [13,14].

This study aimed to investigate the chemical composition, antioxidant potential, and the diuretic effect of three doses of aqueous extract of fresh bee pollen (AEFBP) collected from Morocco in normal rats.

## Materials and Methods

### Ethical approval

Ethical approval was obtained from Sidi Mohamed Ben Abdellah University in Fez, under the responsibility of the Animal Facility and the Laboratory of Natural Substances, Pharmacology, Environment, Modeling, Health and Quality of Life, University of Sidi Mohamed Ben Abdellah, Fez, Morocco (USMBA-SNAMOPEQ 2017-03). The experiments were conducted in accordance with the accepted principles outlined in the “Guide for the Care and Use of Laboratory Animals” prepared by the National Academy of Sciences and published by the National Institutes of Health, and all efforts were made to minimize animal suffering and the number of animals used.

### Chemical characterization by liquid chromatography with diode array detection coupled to electrospray ionization tandem mass spectrometry (LC/DAD/ESI-MS^n^)

#### Bioactive compounds extraction

Fresh bee pollen was obtained from a professional beekeeper from the region of Larache, Morocco, between March and May 2015. The bioactive compounds were extracted by mixing 2 g of fresh bee pollen with 15 mL of ethanol/water (70/30) at 70°C. After 30 min of stirring, the mixture was separated by filtration with Whatman No. 5 filter paper, and the extract was kept at 5°C until analysis Carpes *et al*. [15] by LC/DAD/ESI-MS^n^ using Dionex UltiMate 3000 instrument (Thermo Fisher Scientific, San Jose, CA, USA).

#### Preparation of the AEFBP

To assess the antioxidant and diuretic activity, AEFBP was prepared according to Tohamy *et al*. [16] with a slight modification. Three concentrations, namely, 100, 250, and 500 mg were each added to 10 mL of distilled water and then mixed and sonicated for 30 min. Each solution was stored to stand overnight in darkness and then filtered through a Whatman No. 1 filter paper. The filtrate was stored at −20°C until further use.

### Total polyphenolic content estimation

According to previous studies [17,18], with a slight modification, the total phenolic content in AEFBP was estimated using the reaction with Folin–Ciocalteu reagent; 300 μL of 0.2 N Folin–Ciocalteu solution and 250 μL (75 g/L) of Na_2_CO_3_ were added to 50 μL of fresh bee pollen solution. Then, at room temperature, the mixture was incubated for 2 h, and their absorbance was read at 760 nm. Total polyphenol content is expressed as milligram of gallic acid equivalent per gram (GAE/g).

### Flavonoid content determination

As described by Miguel [19], the amount of flavonoid content was estimated using 500 μL of sample or standard mixed with 500 μL of 2% AlCl3. After 1 h of mixture incubation at room temperature, their absorbance was measured at 420 nm. The results were expressed as milligram quercetin equivalent per gram using a quercetin standard curve (QE/g).

#### Estimation of total antioxidant activity by phosphomolybdate assay

Based on Pilar Prieto’s method, the total antioxidant activity of AEFBP was estimated using the phosphomolybdenum method [20]. The total antioxidant activity of AEFBP was expressed as milligram of ascorbic acid per gram using a calibration curve (mg AAE/g of fresh pollen).

#### Determination of 2,2-diphenyl-1-picrylhydrazyl (DPPH) free radical scavenging activity

To determine the DPPH radical scavenging capacity of AEFBP, 25 μL of pollen solution (different decreasing dilutions were prepared) were mixed with 825 μL of DPPH (0.1 mM).

Then, the mixture was incubated in darkness for 1 h at ambient temperature. The absorbance was measured at 517 nm. This absorbance was then used to calculate IC_50_, the concentration of AEFBP required to scavenge 50% of the DPPH free radicals [21].

### Determination of reducing power assay

The capacity of AEFBP to reduce iron ions was monitored according to the procedure by Miguel *et al*. [19] with minor modifications; 50 μL of pollen solution (different dilutions were used) was added to 250 μL of potassium buffer (0.2 M) and 250 μL of ferricyanide (1%). After 25 min incubation in a water bath (50°C), 200 μL of 10% trichloroacetic acid, 200 μL of distilled water, and 120 μL of ferric chloride (0.1%) were added to the reaction solution. The resulting absorbance at 700 nm was used and compared with an ascorbic acid standard curve.

### Reference chemicals

All chemicals used in the antioxidant activity and the dosage of the phenolic compounds were provided by Sigma-Aldrich Corporation, Fallavier, France.

### LC/DAD/ESI-MS^n^ analysis

A Dionex UltiMate 3000 instrument was the ultrapressure liquid chromatography used for LC/DAD/ESI-MS^n^ analyses (Thermo Fisher Scientific, San Jose, CA, USA) connected to a diode array and attached to a mass detector. The chromatography was conducted with a C18 column from Macherey-Nagel Nucleosil (250 mm × 4 mm id; particles diameter of 5 mm endcapped) with a temperature kept constant at 30°C. The conditions applied in the liquid chromatography were based on a previous work [18]; 1 mL/min was used as the flow rate, and 10 μL was set as the volume of injection. The final spectra data were accumulated in the wavelength interval of 190-600 nm.

The LTQ XL linear ion trap mass spectrometer (Thermo Fisher Scientific, CA, USA) was set in the negative ion mode. An ESI source was used on the spectrometer with the following parameters: Source, tube lens, and capillary voltage at 5 kV, −20 V, and −65 V, respectively; 325°C as the temperature of the capillary; and N_2_ used as the sheath and auxiliary gas at a flow rate of 50 and 10 (arbitrary units), respectively [22].

The mass spectra acquisition was made in a full range of 100-1000 m/z. To assess the fragmentation pattern, it was important to run a data-dependent scan by deploying collision-induced dissociation (CID), set at 35 as the collision energy of the CID cell (normalized). All data acquisition was gathered using the Xcalibur^®^ software (Thermo Fisher Scientific, San Jose, CA, USA).

Chemical standards were used to find the identity of the phenolic compounds and, by comparison, the chromatographic characteristics together with its spectrophotometric behavior on UV and the information from mass spectrometry (Thermo Fisher Scientific, San Jose, CA, USA). However, when standards were not commercially available, the structural information was confirmed using UV data and MS fragmentation patterns as previously reported in the literature [22].

Three standards were used for quantification, namely, caffeic acid (0.002-0.35 mg/mL; y = 3 × 10^7^ × − 78726; R^2^ = 0.999), kaempferol (0.01-1 mg/mL; y = 9 × 10^7^ × − 176949; R^2^ = 0.999), and chrysin (0.005-1 mg/mL; y = 2 × 10^7^ × −247019; R^2^ = 0.999). All compounds were quantified using the calibration curve of the structurally closest standard, and the final result was given in equivalent terms. Each value resulted from three different assays and is expressed as mg/g of fresh pollen.

### Experimental animals

This study was conducted on normal male Wistar rats weighting 200±40 g each. The animals were housed in the Animal House-Breeding Center of Biology Department in the Faculty of Science and Technology of Fez, Morocco, in 25°C±2°C, and the light imposed from 6 to 18 h. Rats received daily water and *ad libitum* food.

### Experimental design

Every single rat was placed in a metabolic cage for 48 h before starting the experiment. Five groups with four rats each were used: The first was a control group administered with distilled water (10 mL/kg body weight), the second group was treated by oral furosemide (10 mg/kg body weight), the third group received 100 mg/kg body weight of AEFBP, the fourth group received 250 mg/kg body weight of AEFBP, and the fifth group received 500 mg/kg body weight of AEFBP for 30 days. Urinary samples were collected in graduated cylinders on days 0, 7, 14, 21, and 30 of treatment to assess water excretion and then filtered, centrifuged, and stored for analysis of urinary sodium, chloride, potassium, creatinine, and urea levels.

The blood samples were taken at the end of the treatment using a retro-orbital puncture with diethyl ether on animals slightly asleep [23]. Then, the plasma was separated by centrifugation at 10,000 × g for 10 min and stored at −20°C for analysis of sodium, potassium, chloride, creatinine, and urea [24]. Creatinine clearance was also calculated at day 30 according to the following formula:

Clearance (mL/min) = (U_crea_ × P_crea_)/V

U_crea_: Urinary concentration of creatinine

P_crea_: Plasma concentration of creatinine

V: Urinary volume (mL/min)

The osmolar clearance (C_osm_) was also calculated from urine volume (V), plasma osmolarity (P_osm_), and urinary osmolarity (U_osm_) using the following equation: C_osm_ = (V × U_osm_) × 1/P_osm_. Then, free water clearance (C_H2O_) was calculated according to the following equation: C_H2O_ = V × (1 − U_osm_) × 1/P_osm_. Free water reabsorption (T_CH2O_) was assessed using the following formula: T_CH2O_ = −(C_H2O_).

### Reference drug

Furosemide (Lasilix, Pharma 5, Morocco) was used as the reference drug.

### Statistical analysis

GraphPad Prism 5 software was used to compare all groups using the one-way analysis of variance followed by *post hoc* Tukey’s multiple comparison test. The results were expressed as mean±SEM [25].

## Results

### Chemical characterization by LC/DAD/ESI-MS^n^

**Figure-1:**
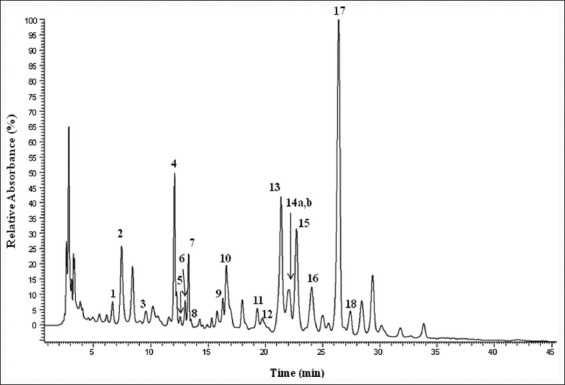
Chromatographic profile of pollen sample obtained at 280 nm by LC/DAD/ESI-MS^n^.

The chromatographic profile of Moroccan fresh bee pollen recorded at 280 nm obtained using LC/DAD/ESI-MS^n^ (Figure-1). Nineteen compounds were identified and reported, including 10 flavonols derivatives, mainly quercetin, isorhamnetin, and kaempferol glycosides; a flavone; luteolin; a phenolic acid; ellagic acid; and seventri-substituted spermidines (Table-1). The quantification of the compounds was made through the chromatogram obtained at 280 nm and using the calibration curves of the phenolic compound whose structure is very close, and its UV spectrum is similar when the standard was not found.

**Table-1:** Bioactive compounds identified and quantified by LC/DAD/ESI-MS^n^ in AEFBP.

Peak	t_R_ (min)	λ_max_ (nm)	(M-H)^-^*m/z*	MS^n^ (% base peak)	Proposed compound	mg/g fresh pollen
1	6.7	264,354	625	MS^2^[625]: 317 (100)	Myricetin-3-*O*-rutinoside^a,c^	0.27±0.00
2	7.5	257,354	625	MS^2^[625]: 445 (85), 300 (92), 301 (100), 271 (17)	Quercetin-diglucoside^a,d^	0.70±0.00
3	9.6	257, 354	609	MS^2^[609]: 301 (44), 300 (100)	Quercetin-3-*O*-rutinoside^a,b,e^	0.23±0.00
4	12.1	266, 347	593	MS^2^[593]: 285(100)	Kaempferol-3-*O*-rutinoside^a,b,e^	0.78±0.00
5	12.2	255, 354	623	MS^2^[623]: 315 (100), 314 (67), 300 (23)	Isorhamnetin-3-*O*-rutinoside^a,b,e^	0.24±0.00
6	13.01	255, 355	609	MS^2^[609]: 315 (100)	Isorhamnetin-*O*-pentosyl-hexoside^a,c^	0.24±0.00
7	13.3	266, 350	635	MS^2^[635]: 285 (33), 284 (100)	Kaempferol-diglucuronide^a^	0.42±0.01
8	13.5	255, 354	477	MS^2^[477]: 462 (45), 314 (18), 315 (100), 300 (13), 299 (12)	Isorhamnetin-3-*O*-glucoside^a,c^	0.16±0.01
9	16.3	256, 349	447	MS^2^[447]: 301 (100)	Quercetin-3-*O*-rhamnoside^a,b,e^	0.32±0.01
10	16.6	253, 367	301	MS^2^[301]: 301 (100), 257 (5), 255 (5), 229 (11)	Ellagic acid^a,b,d^	0.17±0.00
11	19.3	298, 308	614	MS^2^[614]: 494 (24), 478 (100). 468 (5), 452 (78), 358 (18); MS^3^[478]: 358 (100); MS^4^[284]: 315	*N^1^-p-*coumaroyl*-N^5^, N^10^-*dicaffeoylspermidine^a,d,f,g^	0.07±0.00
12	19.7	298, 309	598	MS^2^[598]: 478 (100), 436 (11), 358 (16); MS^3^[478]: 358 (100); MS^4^[358]: 315	*N^1^, N^10^-di-p*-coumaroyl-*N^5^*-caffeoylspermidine^a,d,f,h^	0.06±0.00
13	21.4	254, 268sh, 348	285	MS^2^[285]: 285 (100), 241 (27), 217 (13), 175 (18)	Luteolin^a,b,e^	0.49±0.00
14a	22.1	256, 355	315	MS^2^[315]:300 (100); MS^3^[300]: 271 (100), 255 (4), 151 (<1)	Quercetin-3-methyl-ether^a,e^	0.42±0.00
14b		299, 310	598	MS^2^[598]: 478 (41), 462 (100), 452 (39), 342 (13); MS^3^[462]: 343	*N^1^, N^5^-di-p*-coumaroyl-*N^10^*-caffeoylspermidine^a,d,f,h^	0.10±0.00
15	22.7	294, 308sh	582	MS^2^[582]: 462 (100), 436 (9), 342 (7); MS^3^[462]: 342	*N^1^, N^5^, N^10^-tri-p*-coumaroylspermidine^a,d,f,h^	0.32±0.00
16	24.1	291, 308sh	582	MS^2^[582]: 462 (100), 436 (10), 342 (6); MS^3^[462]: 342	*N^1^, N^5^, N^10^-tri-p*-coumaroylspermidine^a,d,f,h^	0.20±0.00
17	26.4	299, 307	582	MS^2^[582]: 462 (100); MS^3^[462]: 342	*N^1^, N^5^, N^10^-tri-p*-coumaroylspermidine^a,d,f,h^	0.89±0.02
18	27.4	299, 308	582	MS^2^[582]: 462 (100), 436 (10), 342 (6); MS^3^[462]: 342	*N^1^, N^5^, N^10^-tri-p*-coumaroylspermidine^a,d,f,h^	0.10±0.00

a Confirmed with MS^n^ fragmentation;

b Confirmed with standard;

c Confirmed with references: Sobral *et al* [30] Kite *et al*. [38];

d Falcão *et al*. [22];

e Kalaycıoğlu *et al*. [18];

f Bassard *et al*. [9];

g Ares *et al*. [26];

h Sun *et al.* [28]. AEFBP=Aqueous extract of fresh bee pollen

Among the quantified compounds, the *N*^1^,*N*^5^,*N*^10^*-*tri-*p*-coumaroylspermidine (peak 17; 0.89 mg/g of fresh pollen) was present in the highest amount, followed by the flavonols kaempferol-3-*O*-rutinoside (0.78 mg/g of fresh pollen, peak 4), and quercetin diglucoside (peak 2; 0.70 mg/g of fresh pollen). With a concentration of 3.78 mg/g of fresh bee pollen, the flavonols derivatives were the most abundant among the different classes, whereas the spermidine derivatives comprise 1.75 mg/g of fresh pollen (Table-1).

### Antioxidant activity

Table-2 presents the quantification results of total polyphenols, flavonoids, and antioxidant properties of AEFBP. Total antioxidant capacity was in the order of 56.92±0.21 mg AAE/g of fresh pollen. In terms of total polyphenols, the sample contained 44.96±0.51 mg GAE/g; however, total flavonoids were in the order of 2.73±0.07 mg QE.

**Table-2:** Phenols, total flavonoids, and antioxidant activity of AEFBP.

Extract/Standards	DPPH IC_50_ (mg/mL)	Ferric reducing power IC_50_ (mg/mL)	Total phenolics (mg GAE/g)	Total flavonoids (mg QE/g)	Total antioxidant capacity (mg AAE/g)
AEFBP	0.39±0.13	0.54±0.53	44.96±0.51	2.73±0.07	56.92±0.21
BHT	0. 021±0.01	-	-	-	-
Ascorbic acid	-	0.03±0.07	-	-	-

AEFBP=Aqueous extract of fresh bee pollen, BHT=Butylated hydroxytoluene

Regarding free radical scavenging of DPPH and ferric reducing capacity tests, results were expressed as IC_50_ (mg/mL). All tests suggested that AEFBP showed a high scavenging capacity against DPPH (0.39±0.13 mg/mL) and ferric reducing power (0.54±0.53 mg/mL).

### Effect of AEFBP on urinary flow

Urinary excretion was measured at days 1, 7, 15, 21, and 30 of treatment. According to the results presented in Table-3, urine output did not change significantly after treatment with distilled water. By contrast, oral administration of the AEFBP at the dose of 100, 250, and 500 mg/kg body weight showed a significant increase (p<0.05) in urinary output dose-dependently.

**Table-3:** Urine volume (mL) with daily oral administration of pollen, furosemide, and distilled water (mean±SEM).

Groups	Urine volume (mL/24h)				

	Day 0	Day 7	Day 15	Day 21	Day 30
Control	5±0.75	5.5±2.32	4.5±0.24	5.25±0.75	5.5±0.34
Furosemide	4.25±0.4	7.5±0.23**	9±0.18***	12.75±0.24***	13.25±0.32***
Pollen-100 mg	4.5±0.22	7±0.42	16±0.32***^+++^	19±0.67***^+++^	20.5±0.87***^+++^
Pollen-250 mg	4.25±0.54	9±0.87***	8±1.56***^+++^	19.5±1.67***^+++^	20.5±1.78***^+++^
Pollen-500 mg	4.53±0.78	10±1.13***^++^	18.5±1.65***^+++^	20±1.78***^+++^	22.5±1.92***^+++^

*Comparison between normal group and all groups. ^+^Comparison between Furosemide group and pollen groups.

** p<0.01,

*** p<0.001,

+++ p<0.001

Treatment with 100 mg/kg of AEFBP induced a significant elevation of urinary output from day 7 (p<0.01). This increase continued until the last day of treatment. After 30 days, urinary output increased from 4.5±0.22 to 20.5±0.87 mL/24 h (267% increment) for the dose of 100 mg/kg body weight. With 250 mg/kg body weight, urinary output increased from 4.25±0.54 to 20.5±1.78 mL/24 h (273% increment). However, the highest dose (500 mg/kg body weight) induced the highest effect with 309% ­increment; urine output in this group increased from 4.53±0.78 to 22.5±1.92 mL/24 h, whereas furosemide induced a very significant increase in urine output (p<0.001) compared with the distilled water group, but this increase remained less important compared with pollen at different doses.

### Effect of AEFBP on urinary electrolyte excretion

The following data summarize the results of the effects of AEFBP and furosemide on urinary clearance of sodium, chloride, and potassium (Table-4). Furosemide and the three doses of AEFBP showed a significant (p<0.001) increase in urinary clearance of sodium, chloride, and potassium compared with the baseline. However, sodium excretion was dose-dependent, in the order of 180.5±3.4 mmol/L for the first dose (100 mg/kg body weight), 347±6.6 mmol/L for the second dose (250 mg/kg body weight), and 410±8.6 mmol/L for the last dose (500 mg/kg body weight). For furosemide, the increase was in the order of 147.8±5.9 mmol/L.

**Table-4:** Effect of oral administration of AEFBP, furosemide, and distilled water on urinary excretion of creatinine, urea, sodium, potassium, and chloride in normal rats (mean±SEM).

Groups	Urea (g/L)	Creatinine (mg/L)	Urinary electrolytes (mmol/L)			Saluretic index		
	
			Sodium	Potassium	Chloride	Sodium	Potassium	Chloride
Control	12.89±3.12	47±2.7	108±12.7	62.90±2.32	99.0±3.5	1	1	1
Furosemide	25.76±2.12***	53.4±1.9*	147.80±5.9*	160.8±4.11	198±4.9	1.36***	2.55***	2***
Pollen-100 mg	27.16±2.12***	59.6±2.42***^+^	180.5±3.4***^+^	158.5±4.21***^+++^	224.5±5.6***^+++^	1.67***^+++^	2.51***^+++^	2.51***^+++^
Pollen-250 mg	26.89±2.43***	60.2±1.45***^++^	347±6.6***^+++^	181.15±4.32***^+++^	310±7.2***^+++^	3.21***^+++^	2.87***^+++^	3.13***^+++^
Pollen 500 mg	29.85±3.23***	62.8±3.29***^++^	410±8.6***^+++^	238.3±5.21***^+++^	312±6.45***^+++^	3.79***^+++^	3.78***^+++^	3.15***^+++^

* Comparison between normal group and all groups.

+ Comparison between Furosemide group and Pollen groups

*** p<0.001,

+++ p<0.001. AEFBP=Aqueous extract of fresh bee pollen

Moreover, oral administration of AEFBP for 4 weeks caused a significant increase in kaliuresis compared with the distilled water group. For the first dose (100 mg/kg body weight), this increase was in the order of 158.5±4.21 mmol/L, 181.15±4.32 mmol/L for the second dose (250 mg/kg body weight), and 238.3±5.21 mmol/L for the last dose (500 mg/kg body weight). Treatment with furosemide also induced an increase of kaliuresis (160.8±4.11 mmol/L) compared with the control group (62.90±2.32 mmol/L).

Similarly, with urinary potassium, urinary chloride significantly increased after gavage by AEFBP for 30 days. This excretion is in the order of 99.0±3.5 mmol/L in the control group, 224.5±5.6 mmol/L in the treated group (100 mg/kg body weight), 310±7.2 mmol/L in the second group (250 mg/kg body weight), and 312.0±6.45 mmol/L in the last group (500 mg/kg body weight), whereas furosemide group excreted 198.0±4.9 mmol/L of chloride ions (Table-4).

### Effect of AEFBP on creatinine clearance

Fresh bee pollen and furosemide caused a significant increase in creatinine clearance on the last day of treatment when compared with the initial value and the control group. This increase is more significant in the groups treated with pollen at different doses compared with the furosemide group, and the highest effect was assigned to the higher dose (Table-5).

**Table-5:** Effect of oral administration of AEFBP, furosemide, and distilled water on plasma on creatinine clearance (mL/min) in normal rats (mean±SEM).

Groups	Day 1	Day 30
Control	0.032±0.002	0.035±0.001
Furosemide	0.027±0.001	0.096±0.002***
Pollen-100 mg	0.029±0.001	0.163±0.001***^+++^
Pollen-250 mg	0.027±0.001	0.171±0.001***^+++^
Pollen-500 mg	0.029±0.001	0.196±0.003***^+++^

*Comparison between the normal group and all groups. ^+^Comparison between the Furosemide group and Pollen groups. Saluretic index = test mmol/L/control mmol/L. AEFBP=Aqueous extract of fresh bee pollen

### Effect of AEFBP on plasma electrolytes levels

Compared with the control group, administration of AEFBP for 4 weeks caused no significant changes in plasma concentrations of creatinine, urea, potassium, chloride, and sodium. However, furosemide has led to a significant decrease in plasmatic chloride and potassium levels (Table-6).

**Table-6:** Effect of oral administration of AEFBP, furosemide, and distilled water on plasma electrolyte levels (mean±SEM).

Groups	Plasma electrolytes (mmol/L)			Urea (g/L)	Creatinine (g/L)

	Sodium	Potassium	Chloride		
Control	164.5±6.36	5.7±0.23	102.5±5.12	0.5±0.02	5±0.26
Furosemide	160.2±7.71	3.9±0.3***	99.75±4.1	0.49±0.02	5.1±0.31
Pollen-100 mg	156±6.41	5.5±0.29^+++^	104±2.82	0.52±0.03	5.2±0.30
Pollen-250 mg	163.5±7.67	5.4±0.27^+++^	106.5±3.12	0.52±0.02	5±0.30
Pollen-500 mg	167±8.48	5.2±0.2^+++^	111±2.56	0.51±0.04	5±0.21

*Comparison between the normal group and all groups. ^+^Comparison between Furosemide group and Pollen groups.

*** p<0.001,

+++ p<0.001. AEFBP=Aqueous extract of fresh bee pollen

### Effect of AEFBP on osmolarity and free water clearance

Compared with the control group, after 4 weeks of treatment, fresh bee pollen and furosemide caused a significant increase in osmolar clearance, free water clearance, and urine osmolarity (p<0.001). This increase was more significant in the groups treated with different doses of AEFBP compared with the furosemide group, and the highest effect was assigned to the higher dose. Therefore, no significant effect of pollen or furosemide was found on plasma osmolarity (Table-7).

**Table-7:** Effect of oral administration of AEFBP, furosemide, and distilled water on plasma osmolarity, urine osmolarity, osmolar clearance, and clearance of free water on day 30.

Groups	Plasma osmolarity (mOsm/kg)	Urine osmolarity (mOsm/kg)	Cosm (μL/min)	CH_2_O (μL/min)	TCH_2_O (μL/min)
Control	329±12.67	555.8±38.27	6.45±1.31	−2.63±0.31	2.63±0.31
Furosemide	320.4±22.54	1044.8±34.71***	30±9.82**	−20.8±9.31***	20.8±9.31***
Pollen-100 mg	312±17.74	1128.9±31.92***	51.51±16.22***^++^	−37.27±8.74***	37.27±8.74***^+^
Pollen-250 mg	327±21.62	1502.6±54.11***^+++^	65.41±14.71***^+++^	−51.18±9.87***^+++^	51.18±9.87***^+++^
Pollen-500 mg	334±19.32	1792.1±61.62***^+++^	83.84±11.21***^+++^	68.21±12.31***^+++^	68.21±12.31***^+++^

*Comparison between the normal group and all groups.

+ Comparison between the furosemide group and Pollen groups. AEFBP=Aqueous extract of fresh bee pollen

## Discussion

This study aimed to assess the antioxidant and diuretic effect of oral administration of AEFBP in normal Wistar rats. Several recent scientific studies have discussed the importance of phenolic compounds in several pathologies because of their intensive range of properties, such as antimicrobial, antihyperlipidemic, anti-inflammatory, immunoregulatory, and antidiabetic activities. However, the antioxidant property is the major activity revealed in these compounds because of the flavonoids present largely in human alimentation [26,27].

Fresh bee pollen has a large amount of phenolic bioactive compounds such as flavones and flavonols, which are the particularly important constituents from bee plant-derived products. However, climatic conditions such as soil type, weather, and geographic and botanical origins are factors influencing chemical bee pollen composition and other factors such as beekeeper activities [28,29].

### Chemical characterization by LC/DAD/ESI-MS^n^

The chemical analysis of the Moroccan fresh bee pollen sample allowed the detection of 19 compounds, including flavonol and spermidine ­derivatives (Table-1 and Figure-1). The positive confirmation of quercetin-3-*O*-rutinoside (peak 2, *m/z* 609), kaempferol-3-*O*-rutinoside (peak 4, *m/z* 593), quercetin-3-*O*-glucoside, isorhamnetin-3-*O*-rutinoside (peak5, *m/z* 623), ellagic acid (peak 10, *m/z* 301), and luteolin (peak 13, *m/z* 285) was made considering retention time, UV-Vis profile, and MS pattern of the commercial standards.

For the remaining compounds, it was important to interpret the fragmentation pathways detected in MS^n^ spectra with that available in the literature and combine it with the spectral information from UV. Identification of sugar moieties of the flavonoid was made considering that sugar is most common in nature and hence assigned to pentosides, glucosides, rutinosides, and glucuronides.

It is important to note a large number of glycosylated flavonols in these Moroccan fresh bee pollen samples. The MS^2^ of the [M-H]^−^ at *m/z* 625 (peak 1) showed an ion at *m/z* 317 (−308 Da), compatible with a loss of a deoxyhexosyl-hexoside from a myricetin moiety, most probably in the C3 position, being assigned the compound as myricetin-3-*O*-rutinoside [30]. Peak 2 and 14a were identified as quercetin derivatives because of the production observed at *m/z* 301 and the characteristic UV spectrum with UV_max_ at 257 and 354 nm. MS^2^ spectrum of compound 2, with *m/z* 625, presented the main loss of a hexoxyl-hexoside residue (*m/z* 301; −324 Da), allowing the identification as a quercetin-hexosyl-hexoside, most probably a diglucoside moiety [31]. Peak 14a was assigned as quercetin-3-*O*-methyl-ether, where it was possible to observe a significant [M-H-CH_3_]^−.^ product ion typical of methylated flavonoids [22].

The fragmentation pathway shown by the main production in peaks 6 and 8 (*m/z* 315) and the UV data (255 and 355 nm) were compatible with isorhamnetin. In peak 6, MS^2^ spectrum indicated a loss of 294 Da corresponding to a pentosyl-hexoside linked together, whereas in peak 8, the production was formed by the loss of 162 Da, which is indicative of a glucoside unit.

Peak 7 showed a UV_max_ at 266 and 350 nm and a pseudo-molecular ion [M-H]^−^ at *m/z* 636. The MS^2^ produced a fragment at *m/z* 284 with a loss of 351 Da, which is indicative of a diglucuronide moiety linked to kaempferol [32,33]. Among the flavonoids detected, quercetin derivatives are the main compounds (1.89 mg/g of fresh pollen), which are in agreement with the literature where bee pollen is reported to be rich in quercetin derivatives compared with the remaining flavonols [34].

**Figure-2:**
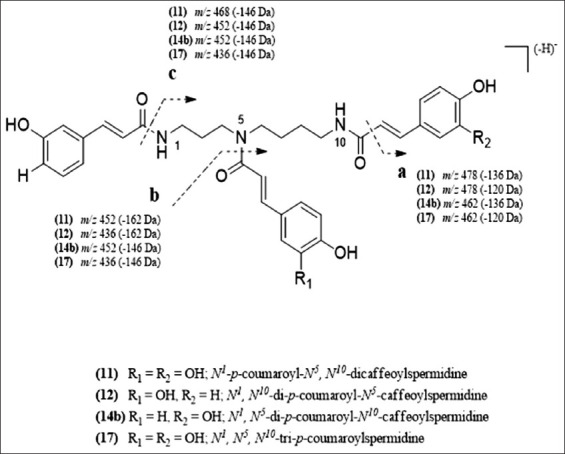
ESI-MS^2^ fragmentation pattern of the spermidine derivatives identified in the Moroccan fresh bee pollen.

Another important group of compounds detected in this sample was spermidines which are phenolamides, that is, N-acylated biogenic amines conjugated with phenolic acids, which are mainly hydroxycinnamic acid amides [9]. The polyamine amino groups can present various degrees of saturation, occurring as mono- or poly-substitution with the same or different hydroxycinnamic acids [8]. Spermidines are the main phenolic constituents of reproductive organs and seeds related throughout the plant kingdom. Several spermidine derivatives were detected in these Moroccan fresh bee pollen samples, including *N*^1^-*p*-coumaroyl-*N*^5^,*N*^10^-dicaffeoylspermidine (peak 11), *N*^1^,*N*^10^-di-*p*-coumaroyl-*N*^5^-caffeoylspermidine (peak 12), *N*^1^,*N*^5^-di-*p*-coumaroyl-*N*^10^-caffeoylspermidine (14b), and *N*^1^,*N*^5^,*N*^10^-tri-*p*-coumaroylspermidine and its three isomers (15, 16, 17, and 18, respectively) (Table-1 and Figure-1). Fragmentation patterns for the identified spermidines are shown in Figure-2, and the identities were tentatively assigned when comparing with the available data in the literature. All the spermidine derivatives showed a UV spectra specific of phenolamides with a UV_max_ at around 298 and 209 nm [8]. The ESI-MS^2^ spectrum of peak 11 indicated a [M-H]^-^ ion at *m/z* 478 attributed to the loss of 136 Da from the caffeic acid residue, formed from the cleavage in the fragmentation pathway (Figure-2a), indicating the substitution of *N*^10^ by a caffeoyl moiety [31]. A major ion at *m/z* 452 (−162 Da) could be assigned to the preferential loss of another caffeoyl residue at N^5^ as previously observed, fragmentation pathway (Figure-2b) [35]. The signal at *m/z*468 could arise from the cleavage of the amide bond between the coumaroyl residue and the spermidine moiety, as shown in the fragmentation pathway (Figure-2c) indicating the substitution of *N*^1^ by another coumaroyl group. The compound in peak 11 was tentatively identified as *N*^1^-*p*-coumaroyl-*N*^5^, *N*^10^-dicaffeoylspermidine. Same fragmentation patterns were observed for the compounds in peaks 12, 14b, and 15-18 (Figure-2) with respective differences correlated with the type of hydroxycinnamoyl moieties, *p*-coumaroyl, or caffeoyl, linked to the different amino groups in the molecules. Peaks 15-18 indicated the same pseudo-molecular ion at *m/z*582, pointing out that they might correspond to different *N*^1^,*N*^5^,*N*^10^-tri-*p*-coumaroylspermidine isomers. The isomer of the latter compound present in peak 17 was one of the most abundant, with a concentration of 0.89 mg/g of fresh pollen, generating the spermidines a total of 1.75 mg/g of fresh pollen. Spermidines, particularly tri-substituted derivatives, are largely present in the reproductive organs of many higher plants, being reported in the pollen of different plant families such as *Malus domestica* [8], *Quercus dentata* [36], *Helianthus annuus* [37], *Arachis hypogaea* [38], *Sambucus nigra* [39], *Hippeastrum x hortorum* [40], and *Ambrosia artemisiifolia* L. [31].

### Antioxidant and diuretic activities

These results showed that AEFBP contains a considerable quantity of total phenols (44.96±0.51 mg GAE/g). Many studies showed that bee pollen harvested from different Turkish regions also contains an important rate of flavonoids and phenolic acids and has a strong antioxidant activity. The highest values of total phenolics were found in chestnut pollens (174.6±120 mg GAE/g) [18]. Table-2 presents a high total flavonoid content and total antioxidant activity in our sample.

We used furosemide as the reference drug to assay the diuretic effect of our sample. As they are largely known, diuretics are used for the treatment of fluid accumulation caused by certain diseases such as high blood pressure and heart or kidney disease by increasing the rate of urine elimination [12,41]. Excretion of Na^+^ is also widely useful in clinical diuretics, which is a major determinant of extracellular fluid volume. Furosemide acts by inhibiting Na^+^-K^+^-2Cl^−^ symporter and, thereby, interfering with their absorption [42].

The three doses of AEFBP used in this study showed a potent effect in increasing the urinary flow rate. The effect was even more important than the standard drug (furosemide). A significant increase in urinary elimination of electrolytes such as sodium, potassium, and chloride was found in a dose-response manner with no effect on plasma electrolytes.

Furthermore, oral administration of AEFBP showed a significant increase in urea elimination and creatinine clearance. Investigating its effect in cases of kidney failure might explore its potential effect to increase creatinine clearance or to alleviate acute/chronic kidney failure.

In general, diuresis is characterized by the occurrence of two events: Augmentation of water excretion (urine output) and an increase in electrolytes elimination, the same that furosemide does by increasing the urinary flow and the sodium elimination by blocking the Na/K/2Cl symporter in the thick ascending branch of the loop of Henle [43,44].

The mechanism involved in the diuretic effect of AEFBP used in this study is not clear yet, but it could have a comparable effect with loop diuretics because they increased urinary elimination of potassium and sodium similar to furosemide. Similarly, it is also known that phenolic compounds and flavonoids can enhance glomerular filtration rate by increasing renal blood flow, favoring a rise in urine formation [41,45,46]. Therefore, the potent diuretic effect of AEFBP might be assigned to the high flavonoid content in our sample. Further studies should be conducted to explore the mechanism of action.

## Conclusion

The composition of Moroccan fresh bee pollen was determined, presenting a high quantity in flavonols and spermidine derivatives. The main flavonols were quercetin diglucoside and kaempferol-3-*O*-rutinoside, whereas *N*^1^,*N*^5^,*N*^10^-tri-*p*-coumaroylspermidine was the main spermidine derivative detected in the ­sample. This study is considered the first to assess the antioxidant power and the effect of AEFBP on diuresis compared with furosemide. The oral administration of three doses of the AEFBP showed a significant increase in the 24 h urine output after treatment. Moreover, the treatment with our extract increased the urine elimination of sodium, potassium, and chloride dose-dependently and caused an increase in urine osmolarity. This strong diuretic activity seems to be directly related to the high antioxidant constituents, such as phenolic compounds largely contained in our extract. Further clinical assays and researches are needed to investigate the diuretic effect Moroccan bee pollen and its medical uses in the treatment of kidney pathologies caused by oxidative stress.

## Authors’ Contribution

BL: Conceptualization, supervision and review of the draft manuscript. AE:Performed experimental studies, and prepared the manuscript draft. AE and NE: Performed the data acquisition and statistical analysis. MV: Performed the LC/DAD/ESI-MS^n^ analysis. SIF and MV: Identified the bioactive compounds. All authors read and approved the final manuscript.
